# The Evaluation of Nerve Growth Factor Over Expression on Neural Lineage Specific Genes in Human Mesenchymal Stem Cells 

**DOI:** 10.22074/cellj.2016.4313

**Published:** 2016-05-30

**Authors:** Yousef Mortazavi, Fatemeh Sheikhsaran, Gholamreza Khamisipour Khamisipour, Masoud Soleimani, Ali Teimuri, Somayeh Shokri

**Affiliations:** 1 Department of Molecular Medicine and Genetics, Faculty of Medicine, Zanjan University of Medical Sciences, Zanjan, Iran; 2 Department of Hematology, Faculty of Allied Medicine, Bushehr University of Medical Sciences, Bushehr, Iran; 3 Department of Hematology, Faculty of Medical Sciences, Tarbiat Modares University, Tehran, Iran; 4 Health Research Institute, Infectious and Tropical Diseases Research Center, Ahvaz Jundishapur University of Medical Sciences, Ahvaz, Iran

**Keywords:** Mesenchymal Stem Cells, Human Nerve Growth Factor, Neural Differentiation, ELISA

## Abstract

**Objective:**

Treatment and repair of neurodegenerative diseases such as brain tumors, spinal cord injuries, and functional disorders, including Alzheimer’s disease, are challenging problems. A common treatment approach for such disorders involves the use of mesenchymal stem cells (MSCs) as an alternative cell source to replace injured cells. However, use of these cells in hosts may potentially cause adverse outcomes such as tumorigenesis and uncontrolled differentiation. In attempt to generate mesenchymal derived neural cells, we have infected MSCs with recombinant lentiviruses that expressed nerve growth factor (*NGF*) and assessed their neural lineage genes.

**Materials and Methods:**

In this experimental study, we cloned the *NGF* gene sequence into a helper dependent lentiviral vector that contained the green fluorescent protein (*GFP*) gene. The recombinant vector was amplified in DH5 bacterial cells. Recombinant viruses were generated in the human embryonic kidney 293 (HEK-293) packaging cell line with the helper vectors and analyzed under fluorescent microscopy. Bone marrow mesenchymal cells were infected by recombinant viruses for three days followed by assessment of neural differentiation. We evaluated expression of NGF through measurement of the NGF protein in culture medium by ELISA; neural specific genes were quantified by real-time polymerase chain reaction (PCR).

**Results:**

We observed neural morphological changes after three days. Quantitative PCR showed that expressions of NESTIN, glial derived neurotrophic factor (*GDNF*), glial fibrillary acidic protein (*GFAP*) and Microtubule-associated protein 2 (*MAP2*) genes increased following induction of NGF overexpression, whereas expressions of endogenous NGF and brain derived neural growth factor (*BDNF*) genes reduced.

**Conclusion:**

Ectopic expression of NGF can induce neurogenesis in MSCs. Direct injection of MSCs may cause tumorigenesis and an undesirable outcome. Therefore an alternative choice to overcome this obstacle may be the utilization of differentiated neural stem cells.

## Introduction

Mesenchymal stem cells (MSCs) are multipotent stem cells that can differentiate to form osteogenic cells, adipocyte cells and chondrocytes. MSCs are derived from multiple tissues, bone marrow and umbilical cord blood (1,2). MSCs have useful features that make them a unique source for cell therapy. These cells can proliferate *in vitro*, easily expand and produce adequate numbers of cells. The ability to express neurotrophins such as nerve growth factor (NGF) and brain derived neural growth factor (BDNF) make them an option for differentiation into neural cells and achieve desired cells for treatment of certain functional tissue defects. These cells can transdifferentiate into several cells under controlled conditions and modulate the immune system when used in the host. Direct injection of MSCs have shown adverse outcomes in hosts, including tumor formation (3) and uncontrolled differentiation into ectopic cells (4,5). It is preferable to generate and propagate specific somatic cells *in vitro* before infusion. There are several procedures for neuronal differentiation of MSCs, including the use of growth factors and co-culture methods (6). 

NGF, the first discovered member of the neurotrophins family, is essential for survival, growth and differentiation of central and peripheral nerves (7,9). NGF is normally expressed in the adult hippocampus and cortex, and provides neurotrophic support to cholinergic neurons. This factor plays a critical role in the regeneration of peripheral nerves after injury. NGF promotes proliferation and differentiation of neural progenitors (10). The administration of recombinant β-NGF protein into injured nerves has been shown to promote nerve repair and enhance functional restoration following nerve damage. Local delivery of NGF is problematic due to its short halflife. In addition, the NGF protein cannot pass through the blood brain barrier (11,12). A useful method for treatment of neuronal damage without injections of NGF is the *in vitro* generation of nerve-like cells and their direct use for tissue repair. 

The present study intended to evaluate the effect of NGF upregulation on expression of specific neural genes. We constructed recombinant replication-deficient lentiviral vectors that expressed human NGF under an eukaryotic promoter in an attempt to induce a neuronal differentiation state in human bone marrow derived MSCs. 

## Materials and Methods

This experimental, descriptive study was conducted on human bone marrow derived MSCs that were transducted by a recombinant lentiviral vector which carried the *NGF* gene. 

### Construction of the recombinant vector

We used a commercially prepared human NGF-β ORF sequence (pCMV Sport6, Open Biosystems). A sequence with 726 nucleotide base pairs was amplified from the ORF by pfu DNA polymerase (Fermentase Thermo Scientific, USA) and specific primers (F: 5ʹ-CTAGTCTAGAGCCA CCATGTCCATGTTG TTC TA CAC-3ʹ, R: 5ʹ-CGC GGATCC TCAG GCTCTT CTCA CA GCC TTCC-3ʹ) that contained Xba1/BamH1 cloning sites, respectively. The polymerase chain reaction (PCR) conditions were as follows: 4 minutes at 95˚C (1 cycle), 10 seconds at 95˚C, 15 seconds at 64˚C (25 cycles), and 20 seconds at 72˚C (5 cycles). After digestion, the amplified ORF was ligated into a pCDH-CMV-MCS-EF1-copGFPCD511B-1 lentiviral vector (System Biosciences, USA) and confirmed by colony PCR and enzymatic mapping. The cloned gene was confirmed by sequencing (CMV promoter primer, 5ʹ-CACGCTGTTTTGACCTCCATAGA-3ʹ). 

### Generation of recombinant viruses

**Fig.1:**
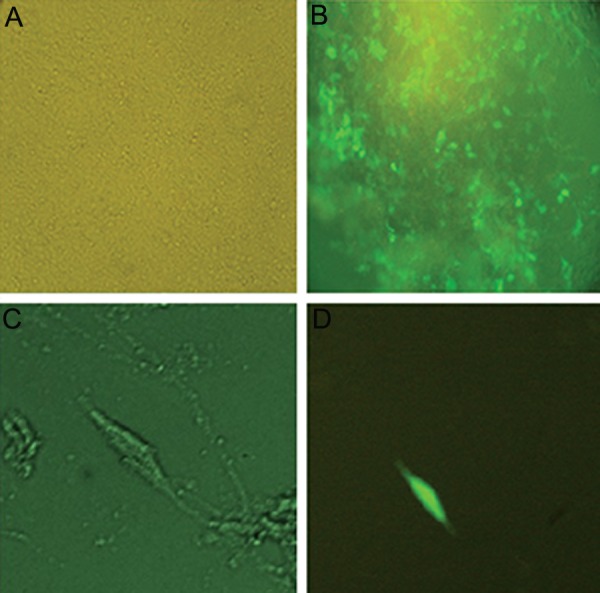
Fluorescent microscopic evaluation that showed expression of green fluorescent protein (GFP) in transduced cells which suggested the existence of recombinant viruses with nerve growth factor (NGF). A, B. Cells at ×10, C. and D. ×40 magnification showed infection of mesenchymal stem cells (MSCs) by recombinant viruses that expressed GFP.

**Fig.2:**
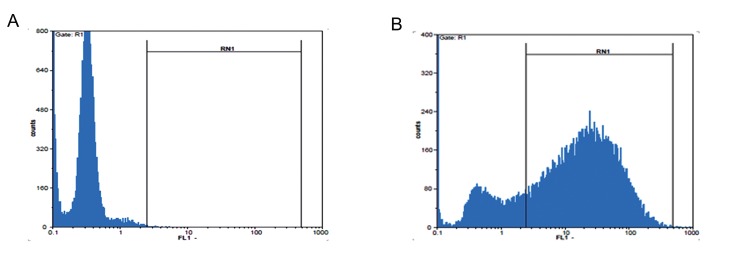
A. Flow cytometric analysis of green fluorescent protein (GFP). Non-infected cells compared to B. 48 hours post-infection which showed a high expression level of protein after transduction of the recombinant viruses that carried the GFP sequence.

We cultured the human embryonic kidney (HEK293) cell line (Pasteur Institute, Iran) in 5% CO_2_ and 37˚C in a humidified incubator in Dulbecco’s Modified Eagles’s Medium (DMEM, Invitrogen, USA) medium that contained 10% fetal bovine serum (FBS) and 1X penicillin/streptomycin (Invitrogen, USA). When the cells reached 80% confluency, we used lipofectamine 2000 (Invitrogen, USA) to simultaneously transfect them with a recombinant plasmid, along with pMD2G and PAX2 plasmids (Addgene, USA) that encoded a VSV-G envelope and packaging genes onto a 10 cm^2^ culture plate. A total of 2×10^5^293T cells/cm^2^ were plated in a 10-cm^2^ plate in 10 ml medium and transfected with 21 µg of transfer plasmid with the human NGF (*hNGF*) gene, 10.5 µg pMD2G plasmid, and 21 µg PAX2 plasmid. The medium was removed approximately 14-16 hours post-transfection and replaced by fresh medium. Cells were collected 24 and 48 hours later, filtered through a 0.45 µm filter and stored at -80˚C. Expressions of the viral genes were monitored by observation of the green fluorescent protein (GFP) under fluorescent microscope (Fig.1). Viral particles were serially titrated and we performed flow cytometric analysis as described by Gueret et al. (13). Briefly, HEK293 cells were infected by different volumes of the collected viruses (10, 50, 100, 150, 250 μl) in 48-well plate. Infected cells were detached by trypsinization at 48 hours postinfection and resuspended in phosphate-buffered saline (PBS, Sigma, USA), then analyzed immediately by flow cytometry (Fig.2). Green fluorescent units (GFU), as indicators of infective viral particles, were calculated according to the following formula: [(1×10^5^seeded cells×% GFP-positive cells)×1000]÷volume of diluted viruses (µl). 

### Culture and expansion of mesenchymal stem cells

Human bone marrow MSCs were kindly gifted by Bonyakhte Stem Cell Research Center (Tehran, Iran). The cells were cultured in low glucose DMEM with 10% FBS and 1X penicillin/streptomycin in a T75 cm^2^ flask at approximately 300×10^3^cells per flask. When cells reached greater than 80% confluency, they were detached by trypsin/ EDTA 0.25% (Sigma, USA), then transferred to 24-well plates. We changed the medium every 48 hours. Viability was more than 95% at the time of the study, as assessed by 3% trypan blue dye (Gibco, Germany). 

### Detection of recombinant nerve growth factor-β

We used an ELISA quantitation kit (Human betaNGF ELISA kit, Ray Biotech, Inc, USA) to measure human NGF levels in culture medium of infected cells at days 4 and 7 according to the manufacturer’s instructions. Purified hNGF protein was used as the standard. Standard and culture extract that contained hNGF were incubated on a plate at room temperature for 2.5 hours. After washing, biotinylated antibody was added and the mixture incubated for one hour at room temperature. Detection was performed by using streptavidin solution followed by TMB detection on an ELISA reader at 450 nm. 

### Transduction of mesenchymal stem cells

We used ultra-centrifuged concentrated viral particles at a Multiplicity of infection (MOI) of 10-15 after pretest experiments. MSCs were seeded in duplicate into 24-well plates in DMEM medium and 5% FBS at a density of 5×10^4^cells per well according to their viability. Recombinant viruses were added to the culture, while shaking, for 2 hours at 37˚C in a CO_2_ incubator. The medium was changed 6 hours after transduction. Differentiation was monitored morphologically and analyzed by RT-PCR and real-time PCR at 24, 48, and 72 hours after infection of the MSCs. 

### Quantitative assessment of gene expressions

We harvested the infected cells were harvested at pre-determined intervals (24 and 96 hours) and extracted the total RNA (Qiazole, Qiagene, USA). Briefly, the infected cells were harvested and centrifuged at 300 g for 5 minutes. Lysis solution was added to the cell pellet with 2 mercaptoethanol (Invitrogen, USA) and vortexed twice for 10-15 seconds each, incubated at room temperature, then after centrifugation at 11000 g for 1 minute, we added chloroform and purified the RNA layer with ethanol. cDNA was synthesized according to the manufacturer’s instructions (First Strand cDNA Synthesis Kit, Qiagene, USA). Expression of specific neural differentiation was quantified by real-time PCR with primers for *NGF, NESTIN, TUBULIN,* Microtubule-associated protein 2 (*MAP2*), Glial fibrillary acidic protein (*GFAP*), BDNF, and Glial derived neurotrophic factor (*GDNF*) as shown in Table 1. The fold difference in gene expression was calculated by the Livak method (2^-Δ∆CT^). Non-transducted cells were used as the control and *GAPDH* as the reference gene. The PCR conditions were 10 minutes at 95˚C (1 cycle) followed by 10 seconds at 95˚C, 15 seconds at 60˚C, and 20 seconds at 72˚C (40 cycles). 

### Statistical analysis

All experiments were performed in triplicate. We used the average values for relative quantification. The results were expressed as mean ± SD and analyzed with SPSS 16 software (SPSS Inc., IBM, USA). Difference between groups was evaluated by Kruskal Wallis and comparison of studied groups for the expression of the desired genes was assessed by the Mann-Whitney test. A P value ≤0.05 was considered significant. 

### Ethical consideration

Zanjan University of Medical Sciences Review Board and Ethics Committee approved this study. Human bone marrow MSCs were kindly gifted and characterized for specific mesenchymal markers and plasticity by Bonyakhte Stem Cell Research Center (Tehran, Iran) at passage-3 postderivation from bone marrow. 

## Results

### Transduction of mesenchymal stem cells 

**Fig.3:**
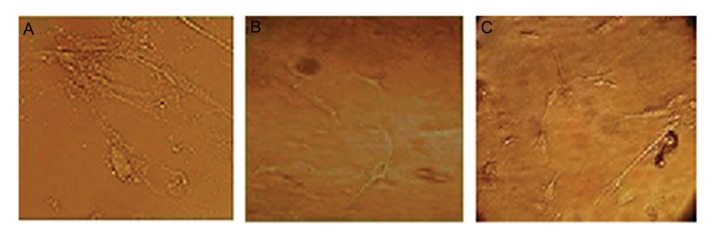
Morphologic changes in mesenchymal stem cells (MSCs) after transduction by recombinant lentiviruses that expressed human nerve growth factor (hNGF). Magnification ×40 showed the appearance of membrane extremities in NGF overexpressed cells at 24 and 48 hours after transduction. A. Non-Transdicted, B. 24 hours, and C. 48 hours post transduction.

Human MSCs were cultured in 24-well plates. We added the virus supernatant when cells reached 70-80% confluency. GFP expression was observed under inverted fluorescent microscope (Fig.1). The percentage of GFP-positive cells after transduction approximated 80% at an MOI of 15. The concentration of viral particles ranged from 5×10^7^ to 8×10^7^ per milliliter. There were morphologic changes observed under the inverted microscope at 24, 48, and 72 hours after transduction (Fig.3). We observed neuronal like transformation with stretched extremities and dendrit- ic shape cells at 48 hours post-treatment.

**Table 1:** Specifications of primers used for quantitative real-time polymerase chain reaction (PCR)


Gene	Sequence(5'-3')	Length(bp)

*GAPDH*	F: CAAATTCCATGGCACCGT C	62
	R: TCTCGCTCCTGGAAGATGGT
*H-BDNF*	F: GTG AATTGA TAATAA ACTGTCCTC	187
	R: TAATTCCAACGCTATCAGAAG
*H-GDNF*	F: GAAATAGAAGGCTGGTGAGTG	78
	R: ACGACAGGTCATCATCAAAG
*H-NGF*	F: GGACCCAATAACAGTTTTACC	75
	R: GAACAACATGGACATTACGC
*NESTIN*	F: GAAGGTGAAGGGCAAATCTG	96
	R: CCTCTTCTTCCCATATTTCCTG
*GFAP*	F: GCAGACCTTCTCCAACCTG	127
	R: ACTCCTTAATGACCTCTCCATC
*MAP2*	F: AGTTCCAGCAGCGTGATG	97
	R: CATTCTCTCTTCAGCCTTCTC
*H-TUBULIN β1 *	F: GATCGGAGCCAAGTTCTG	177
	R: GTCCATCGTCCCAGGTTC


### Quantitative measurement of human nerve growth factor protein in culture

**Fig.4:**
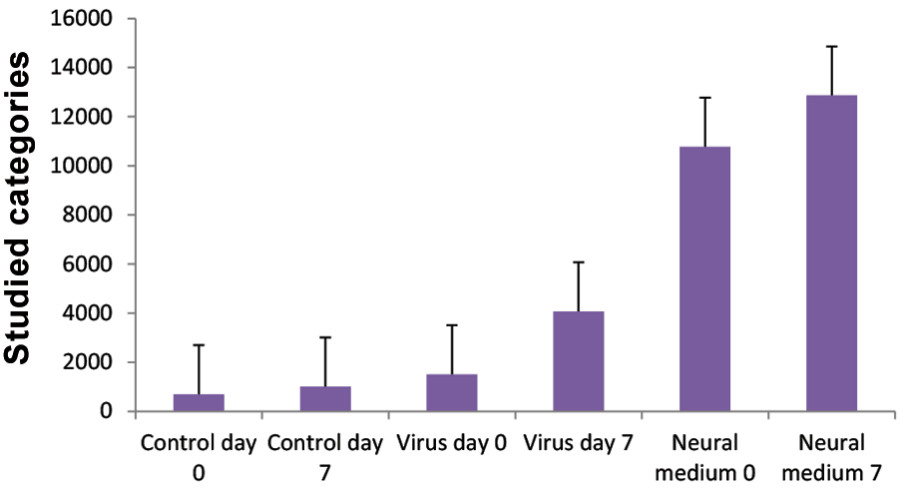
ELISA results of three assayed groups that demonstrated the highest level of nerve growth factor (NGF) in supernatant of cells cultured in medium that contained NGF. There was a significantly high level of NGF expressed in medium of cells infected with recombinant viruses that carried the ectopic *NGF* gene at day 7 compared to the control. Control day 0; Non-treated cells, Control day 7; Non-treated cells at day 7, Virus day 0; Infected cells by recombinant viruses that carried the NGF gene at day 0, Virus day 7; Infected cells by recombinant viruses that carried the *NGF* gene at day 7, Neural medium 0; Cells cultured in commercial neural differentiation medium at day 0 and Neural medium 7; Cells cultured in commercial neural differentiation medium at day 7. Results reported as mean ± SD of three experiments. Error bars indicate SD. P value≤0.05 was considered significant.

The relative levels of hNGF were assessed in supernatant of transformed cells by an ELISA kit at the suggested time. ELISA assay of the culture supernatant revealed stable, high levels of hNGF protein (40.5×10^3^U) under control of the CMV promoter (Fig.4). Because of the shorter half-life of endogenous NGF mRNA, we performed RTPCR at day 4. ELISA was performed at days 0, 4, and 7. Results showed no significant increase in the transducted (1830 pg/ml) and differentiation medium (10340 pg/ml) groups compared to the control group (810 pg/ml) on day 4. However there was a remarkable increase in NGF observed at day 7 for the groups with transducted (4110 pg/ ml) and differentiation (12340 pg/ml) media. 

### Expression of lineage specific genes

**Fig.5:**
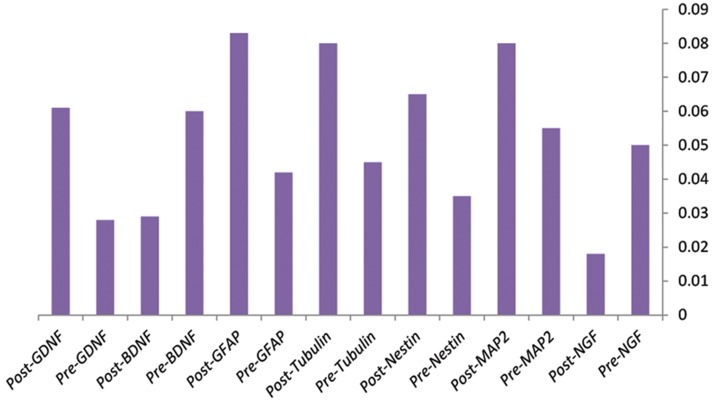
Expression of human nerve growth factor (*NGF*), NES- TIN, Microtubule-associated protein 2 (*MAP2*), Glial fibrillary acidic protein (*GFAP*),* TUBULIN β*, and Brain derived naural growth factor (*BDNF*) genes assessed on day 4 after transduc- tion showed reduction in expression of endogenous NGF and BDNF. Pre; Day 0 before treatment and Post; 96 hours post- virus infection. This result showed the role of Glial derived neurotrophic factor *(GDNF), GFAP, NESTIN,* and *TUBULIN* in the final events of neural differentiation. Results reported as mean ± SD of three experiments.

Transformed cells were harvested 96 hours after transduction with recombinant viruses that contained hNGF. The expressions of neural specific lineage genes were evaluated by quantitative realtime PCR. Our results showed significant over expression of neural genes in transformed cells compared with the control group of non-transducted cells. As shown in Figure 4, despite stable NGF expression in MSCs, ectopic overexpression of NGF alone induced neurogenesis in these cells. We found that the level of endogenous expressions of *NESTIN, MAP2, TUBULIN, GFAP* and *GDNF* genes increased whereas NGF and BDNF expressions down regulated (Fig.5). 

## Discussion

hNGF protein is important for the development and survival of neurons, particularly the sensory neurons. NGF has been shown to promote the division of progenitor cells in rat striatum, as well as induce the differentiation of neurons and their protection under various pathological conditions (14). The concentration of NGF in the central nervous system is very low. The NGF protein has a molecular weight of approximately 13.2 KD, therefore it cannot pass through the blood-brain barrier, which restricts its clinical applications (15,16). Although many studies have sought to restore and repair neural cell related disorders such as Alzheimer’s and conditions such as spinal cord injuries, however direct administration of the NGF protein is not efficient due to the preventive role of the blood brain barrier and immunologic issues (17,18). 

Many studies have shown the positive role of MSCs in treating spinal cord injuries (19,22). Mesenchymal cells can be indefinitely propagated *in vitro* and differentiate to distinct cell types under controlled conditions. Therefore, these cells are suitable sources for cell therapies (22). One method for differentiation of MSCs into neuronal lineage cells is the use of inducing factors such as NGF considering its critical role in neural progression (23,25). 

In the present study, we aimed to demonstrate that despite endogenous expression of NGF, MSCs could differentiate into a neuronal like state through promotion of specific genes of the neural lineage. We found that ectopic over expression of NGF by recombinant lentiviral vector can itself singly induce specific genes of neural lineage. NGF assays in culture medium of the three evaluated groups showed a significant increase in NGF production after 7 days compared to day 0 in cells transducted with viruses that expressed NGF. Although the measured protein in the differentiating medium group was high on day 0, there was no considerable increase after 7 days, which appeared to be due to the existence of NGF in medium rather than its endogenous production by cultured cells. This finding showed efficient production of NGF protein by recombinant viruses and triggered additional expression of endogenous gene. 

According to our results, recombinant viruses that expressed the NGF protein could transdifferentiate MSCs into neuronal like cells without additional growth factors in DMEM medium regardless of endogenous expression. On day 7, most cell populations had transformed and demonstrated neuronal like morphology with small cell bodies that contained extended processes. Our results confirmed the findings of a study that reported morphological transformation 3 hours after induction and maximum differentiation after 7 days (26,27). Quantitative evaluation of specific neural genes 4 days after transduction of recombinant viruses that expressed NGF showed decreased levels of endogenous NGF with significantly increased BDNF and NESTIN. This finding supported the report by Chen et al. regarding the neurogenic roles of NGF, BDNF, and fibroblast growth factor (FGF) (28). Parivar et al. (29) have reported differentiating effects of *TUBULIN* and basic fibroblast growth factor (*bFGF*) on mouse bone marrow MSCs.* NGF* and related genes, i.e, BDNF overexpress during neurogenesis and are essential for this step, however they decrease after the generation of neuronal like cells (30,32). We have observed significant increase in *GDNF, GFAP, NESTIN,* and *TUBULIN* gene expression in cells infected with recombinant viruses (P˂0.05), which showed their critical roles in the final events of neural differentiation. Our finding agreed with previous research on rat bone marrow stromal cells (33). The current study also showed that expression of NESTIN, a protein that expressed during the proliferative state of neural tissues, decreased by day 4. Its expression was transient in the initial step of neurogenesis; after differentiation and proliferation of neural cells this protein was replaced by neural specific filamentous proteins GFAP, TUBULIN, GDNF, and MAP2 (34,35). 

## Conclusion

We believe these lentiviral based viruses circumvent the usual challenges for efficient gene transfer. The recombinant viruses used in the current study could promote efficiently neural differentiating genes only by NGF without any additional factors. However, for clinical use, safer transfer vehicles should be used. Since direct injection of MSCs potentially causes tumorigenesis and undesirable outcomes, the best choice to overcome this obstacle is utilization of differentiated neural stem cells. We believe that the next step should be the use of safer approaches to generate neuronal like cells by non integrating viral vectors which will result in more efficient differentiating methods. 
